# Changes in Indoor Insecticide Residue Levels after Adopting an Integrated Pest Management Program to Control German Cockroach Infestations in an Apartment Building

**DOI:** 10.3390/insects10090304

**Published:** 2019-09-18

**Authors:** Changlu Wang, Amanda Eiden, Richard Cooper, Chen Zha, Desen Wang, Ed Reilly

**Affiliations:** 1 Department of Entomology, Rutgers University, New Brunswick, NJ 08901, USA; 2 Key Laboratory of Bio-Pesticide Innovation and Application of Guangdong Province, Department of Entomology, College of Agriculture, South China Agricultural University, Guangzhou 510642, China; 3 New Jersey Department of Environmental Protection, Trenton, NJ 08625, USA

**Keywords:** insecticide residue, integrated pest management, German cockroach

## Abstract

Insecticide use in homes leads to human exposure to insecticide residues that persist in the environment. Integrated pest management (IPM) programs have been known to be more environmentally friendly for managing German cockroach (*Blattella germanica* L.) infestations, but their effect on indoor insecticide residue levels are not well understood. An IPM program consisting of applying cockroach gel baits and placing insect sticky traps as the primary treatment methods were implemented. Floor wipe samples were collected from the bedroom and kitchen floors of 69 apartments with German cockroach infestations at 0 months and again at 12 months from 49 of the 69 apartments sampled at 0 months. Levels of 18 insecticide residues were measured. The mean insecticide residue concentration per apartment decreased by 74% after 12 months. The number of insecticides detected per apartment decreased from 2.5 ± 0.2 to 1.5 ± 0.2 (mean ± standard error). Indoxacarb residue was only detected in two apartments at 12 months despite the fact that an average of 32 ± 4 g 0.6% indoxacarb gel bait was applied per apartment. IPM implementation can result in significant reduction in the insecticide residue concentrations and number of detected insecticides in floor dust samples.

## 1. Introduction

Residents frequently use insecticides indoors or around homes for control of insect pests [1,2,3]. Since 2001, there has been increased indoor usage of synthetic pyrethroids due to the phase-out of organophosphates in the U.S. [4,5]. A recent survey found 55% of the apartment dwellers in two New Jersey cities used insecticide sprays to manage indoor cockroaches [6]. Among those experiencing bed bug infestations, 59% applied insecticides to control bed bugs in addition to professional pest control services offered by the property management offices [7]. Insecticide use in homes leads to human exposure to insecticide residues that persist in the environment. The main metabolites of pyrethroids have frequently been detected in urine samples from the general population, confirming widespread exposure of children and adults to one or more pyrethroids [8]. Health effects from residential pesticide use include altered fetal growth from prenatal exposure [9], childhood cancer [10,11,12], asthma [13], and decreased fertility [14].

There are a number of studies reporting the prevalence and distribution of indoor insecticide residues [15,16,17,18]. Most of the occupied homes in the U.S. had insecticide residues with permethrin being the most common [17]. Lu et al. (2013) found all 20 sampled homes living in Boston publishing housing development had pesticide contamination in floor wipe or air samples [19]. The most common pesticides were permethrin and cypermethrin. A more recent study found permethrin was detected in 97.5% of the floor wipe samples in residential homes (single homes and apartments) [20]. These studies demonstrated the need for alternative pest control strategies to reduce indoor insecticide use and insecticide residue levels.

The German cockroach (*Blattella germanica* L.) (Blattodea: Ectobiidae) is a very common pest in homes in the U.S. [21]. Many factors, including poor sanitation, lack of proper maintenance of the apartments, poor pest control practices, and natural dispersal of cockroaches between apartments within multi-unit dwellings contribute to the high percentage of apartments with German cockroach infestations [6,21,22]. Worldwide, pyrethrin and pyrethroid insecticides in the form of aerosol sprays, liquid sprays, and total release foggers are commonly used by residents to control cockroaches [2,23,24,25]. However, their efficacy for cockroach management is generally very low due to the prevalence of insecticide resistance among wild cockroach populations [26,27]. A recent study evaluated four different total release fogger products and found they had no significant effect on German cockroach counts after 4 weeks [28]. Gel baits are highly effective in controlling German cockroaches [28,29]. Wang et al. (2009) demonstrated that a community-wide cockroach IPM program reduced cockroach infestation rates by 74% after 12 months [30]. The IPM program included education, de-cluttering of the home, gel bait and/or boric acid dust applications, and follow-up monitoring with sticky traps. Shahraki et al. (2011) compared an IPM program using hydramethylnon gel baits with cypermethrin spray treatment [31]. The IPM program resulted in significantly higher reduction in cockroach infestation compared to cypermethrin spray throughout the 11 weeks of post-treatment period. The published work demonstrates IPM programs, including the use of baits rather than liquid residual insecticides, are effective for sustainable control of cockroach infestations.

Zha et al. (2018) found that after implementing a cockroach IPM program in a low-income community, insecticide residue in the kitchen floor wipe samples decreased by 90% after 7 months [32]. However, the long-term impact of IPM programs on indoor insecticide residue is unclear. The objective of this study was to evaluate the impact of IPM program implementation on indoor pesticide residues over a 12 month period.

## 2. Materials and Methods

### 2.1. Study Site

This study was conducted in a high-rise, low-income building for senior and disabled residents located in Paterson, New Jersey. The thirteen-story building contained 188 units, with 176 one-bedroom and 12 two-bedroom units. A building-wide inspection immediately before this study found 49% with German cockroaches and 8.5% with bed bug (*Cimex lectularius* L.) (Hemiptera: Cimicidae) infestations. Prior to the current study, pests were controlled by a professional pest management service contracted by the management of the apartment building. The contractor only used insecticides and visited the building monthly. For bed bug infestations, Demand^®^ CS (0.03% λ-cyhalothrin, Syngenta Crop Protection, LLC, Greensboro, NC, USA), Suspend^®^ SC (0.06% deltamethrin, Bayer Environmental Science, Research Triangle Park, NC, USA), and Gentrol^®^ (0.07% hydroprene, Wellmark International Brand, Schaumburg, IL, USA) were used. For cockroach infestations, Maxforce^®^ FC Select (0.01% fipronil, Bayer Environmental Science, Research Triangle Park, NC, USA) bait was used. Based on our visual observations, less than one minute was spent per unit and based upon treatment records <0.5 g of bait was applied per unit to kitchen cabinet hinges and the bathroom during each monthly visit. In addition, many residents used over-the-counter sprays to control cockroaches and bed bugs [7]. Most of these insecticide sprays contain various pyrethroids as active ingredients. The self-administered insecticide application frequency and amount were not recorded as most residents were unable to provide accurate information on their insecticide use.

A building-wide inspection was conducted in March 2016 to identify cockroach-infested apartments. Approval from Rutgers University Institutional Review Board (protocol # E16-056) was obtained prior to the study. In each apartment, four Trapper^®^ monitor & insect traps (6.2 × 9.0 cm) (Bell Laboratories Inc., Madison, WI, USA) were placed in every accessed unit. A trap was placed in the cabinet directly under the kitchen sink, next to the stove, next to the refrigerator, and behind the toilet in the bathroom. Traps were checked after approximately 14 days, based upon accessibility to apartments and schedule. A total of 69 apartments with cockroach infestations were identified and the residents agreed to participate in the insecticide residue sampling study. Floor wipe sampling was conducted in these 69 apartments approximately two weeks after obtaining the cockroach trap counts. After 12 months, 49 of these 69 apartments were sampled again in the same manner and from the exact same locations. The other 19 apartments that were not re-sampled at 12 months either did not have detectable insecticide residues at 0 months (17 apartments) or had the lowest residue loading or had a tenant change. The second set of samples were analyzed using the same method as described above.

### 2.2. Floor Wipe Sampling and Insecticide Residue Analysis

Two 1 × 1 ft sample sites (30.48 × 30.48 cm, i.e., 929 cm^2^ floor areas) were selected in the kitchen and bedroom from each apartment. The locations were marked by taking a photo with a 1 × 1 ft wooden frame outlining the sampling spot so that future samples could be taken from the exact same locations. The sampling procedure was same as described in Zha et al. (2018) [32]. A previous study shows that insecticide residue loading from floor wipe samples varies greatly in different areas of a room [33]. We were interested in measuring the insecticide residues from drifted dust, therefore, the exact same locations were sampled to measure the impact of IPM program implementation. There were eight blanks, not wiped but 70% ethanol applied to gauze pads, and eight control wipes that were wiped against clean acrylic tiles (Armstrong World Industries Canada Ltd., Montreal, QC, Canada).

A total of 18 insecticides including chlorfenapyr, chlorpyrifos, fipronil, imidacloprid, indoxacarb, pyriproxyfen, pyrethrin, 10 pyrethroids, and MGK-264 were analyzed (Table S1). The residue concentrations were determined by extracting the wipe samples with 5.0 mL of ethyl acetate followed by 20 min of sonication and then analysis utilizing a low-pressure gas chromatograph/mass spectrometer (GC/MS) technique [34]. An Agilent 5973 Inert GC/MS was configured first with a 3-m non-bonded capillary column with a 0.25 mm inner diameter (ID) attached to the injection port. The non-bonded capillary was connected by means of a glass union to a 10-m J&W capillary column with a 0.53 mm ID with a film thickness of 0.5 µm of Durabond-5^®^ (DB-5). The mega-bore column (0.53 mm ID) was connected through the transfer line to the ion source creating a vacuum in the chromatographic system which improved resolution. The use of a mega-bore column enables a larger injection volume (5 µL) that increased sensitivity and lowered detection levels. The column pressure setpoint was adjusted to 1.00 psi at a constant flow rate setting to produce a linear velocity of 100 cm/sec and a flow rate of 13.2 mL/min. The injector temperature was set to 250 °C and a splitless injection were used with a split vent of 50 mL/min at 0.7 min. The mass spectrometer ion source was set at 230 °C and the temperature of the quadrupole was 150 °C. The transfer line leading to the mass spectrometer was set at 280 °C. Elution of the pesticide analytes from the GC column occurred through the temperature program that ranged from 50 °C to 300 °C over a 7-min chromatographic run. The mass spectrometer operated in electron ionization mode (EI) and scanned from 45 amu (atomic mass units) to 450 amu at a rate of 1.86 scans/sec. Calibration curves for each targeted pesticide analyte were generated each day of sample analysis with reference standards obtained from the EPA national pesticide repository located in Fort Meade Maryland. The mass axis of the spectrometer was calibrated using perfluorotributylamine (FC43) and a system performance check using decafluorotriphenylphosphine (DFTPP) was run each day before any samples.

### 2.3. IPM Program Implementation

All cockroach-infested apartments in the building were treated immediately after the initial floor wipe sampling. Advion^®^ Cockroach Gel Bait (0.6% indoxacarb, Syngenta Crop Protection LLC., Greensboro, NC, USA) was applied thoroughly in kitchens and bathrooms of all apartments with cockroaches. An average of 18 ± 1 g of gel bait was applied per treated apartment. Each placement was approximately 0.1 g. Bait placements were applied in cracks, crevices, interior corners of the cabinets, beside and behind refrigerator and stove. Apartments with >20 cockroaches in traps over 14 days trapping period also received an application of Borid^®^ dust (99% orthoboric acid, Waterbury Companies Inc., Waterbury, CT, USA) behind the refrigerator and under the stove with an average of 2.6 ± 0.4 g per treated unit. Each resident received a one-page pamphlet advising them not to use any insecticides and keep their apartment clean and uncluttered to help with the cockroach control. All apartments were revisited every two weeks for three months then every four weeks and retreated if cockroaches continued to be present. During each visit, cockroach counts were recorded, and four new sticky traps were placed in each apartment. Residents were asked to keep their apartment clean and non-cluttered using the same standards as that described in Wang et al. (2018) [6]. Advion bait was reapplied during biweekly visits if cockroaches were still found in monitoring traps.

In apartments still having cockroaches after 15 weeks, boric acid dust, and baits with active ingredients from other classes of chemistry were applied to combat the possibility of resistance among the remaining cockroaches: Alpine^®^ Cockroach Gel Bait (0.5% dinotefuran, Whitmire Micro-Gen Research Laboratories, Inc., Saint Louis, MO, USA) and boric acid dust during 17 to 22 weeks, Maxforce FC Select during 24 to 28 week, Avert^®^ DF Dry Flowable Cockroach Bait (0.05% abamectin * B1, Whitmire Micro-Gen Research Laboratories, Inc., Saint Louis, MO, USA) during 32 to 44 wk. Treatment stopped after no cockroaches were found for a four week period. The total amount of insecticides used were Advion-918 g, Alpine-245 g, Maxforce FC Select-86 g, Avert-39 g, boric acid dust—213 g. The IPM program had little risk to the occupants because the bait materials and boric acid dust are low in toxicity to mammals and only small amounts of materials were applied in areas that are typically not exposed to human and pets.

There was not a non-intervention control group in this study. The study site had chronic high cockroach infestation rates prior to the study. The 0 and 12-month sampling occurred in the same season. We assumed cockroach infestation levels, pest control practices by residents and building pest control provider would remain similar without intervention, and therefore insecticide residue loading from dust samples would remain stable from 0 to 12 months.

### 2.4. Data Analysis

Paired t-test was used for comparing the number of insecticides by room type when data were normally distributed. Regression analysis was conducted between logarithmically transformed residue concentration per apartment and initial cockroach count. For non-normally distributed data, the Wilcoxon signed-rank test was used. These include differences in the concentration of individual or total insecticide residue between bedrooms and kitchens, number of insecticides between bedrooms and kitchens, changes in insecticide residue concentrations and number of insecticides by room or by apartment over a 12 month period. All analyses were performed using SAS software version 9.3 [35].

## 3. Results

### 3.1. Baseline Insecticide Residues

**Figure 1:**
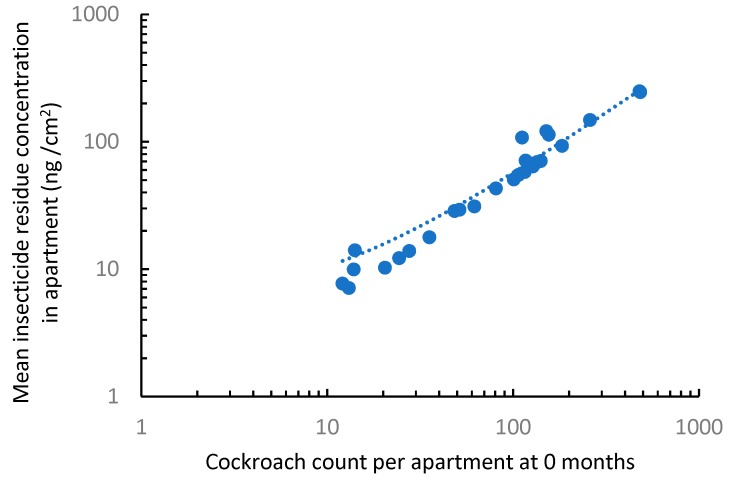
Association between insecticide residue concentration per apartment and initial cockroach count. Those apartments without detected residue and those with cockroach count <10 were excluded because there are many apartments with small counts which make the data not normal even after logarithmic transformation.

**Figure 2:**
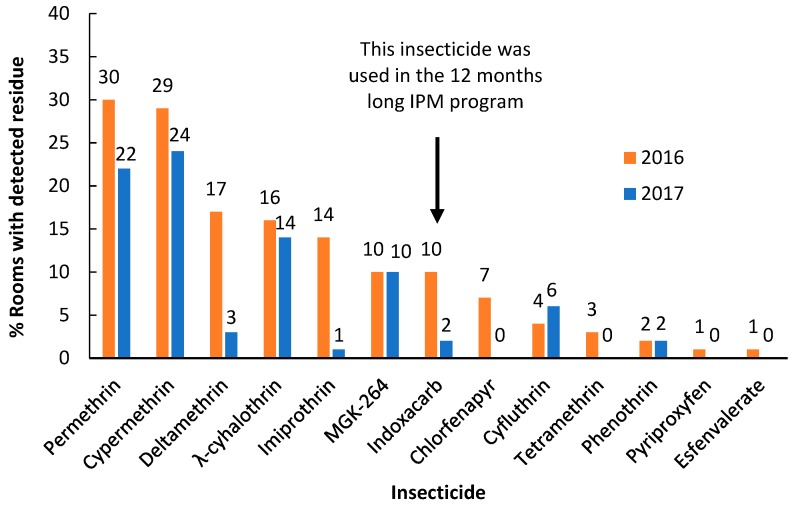
Detection frequency of insecticides in 98 rooms (from 49 apartments) based on floor wipe samples. Only those apartments that were sampled both at 0 and 12 months were included.

**Figure 3:**
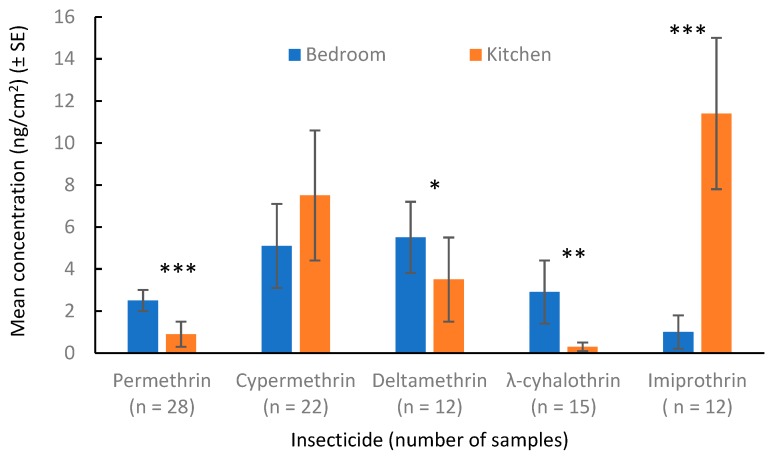
Distribution of the top five insecticides between bedrooms and kitchens in apartments where these insecticides were detected at 0 months (* *p* < 0.05, ** *p* < 0.01, *** *p* < 0.001).

Total cockroach counts in traps per apartment ranged from 1 to 484 in the 69 apartments. No residues were detected in the two types of control samples, indicating the materials used did not contain any insecticide contamination and the sampling procedure did not introduce contaminants. At 0 months, 49 out of the 69 (71%) apartments had detectable insecticide residues. Insecticide residue concentration in apartments was closely correlated with the cockroach counts recorded by the cockroach traps (F = 677.4; df = 1,25; *p* < 0.001; R^2^ = 0.96) (Figure 1). Among the 18 insecticides measured, 13 insecticides were detected at 0 months. Their relative abundance is shown in Figure 2. The top five most frequently detected insecticides and their frequency of detection in rooms were permethrin (30%), cypermethrin (29%), deltamethrin (17%), λ-cyhalothrin (16%), and imiprothrin (14%). Distribution of these insecticides between bedrooms and kitchens in each apartment where these insecticides were detected is shown in Figure 3. Higher concentrations of permethrin, deltamethrin, and λ-cyhalothrin were seen in bedrooms than in kitchens (Wilcoxon signed-rank test, *p* < 0.05). Higher concentrations of imiprothrin were found in kitchens than in bedrooms (Wilcoxon signed-rank test, S = −39, *p* < 0.01). Similar levels of cypermethrin were found between bedrooms and kitchen (Wilcoxon signed-rank test, S = −50, *p* = 0.11).

**Figure 4:**
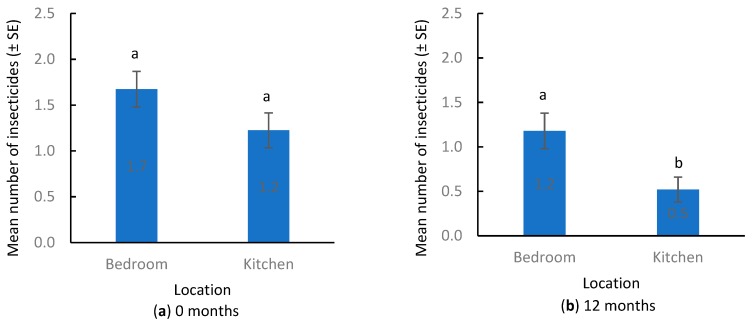
Number of insecticides detected by location. (**a**) 0 months, (**b**) 12 months. Different lowercase letters above the bars indicate significant differences between room type (Wilcoxon signed-rank test, *p* < 0.05).

**Figure 5:**
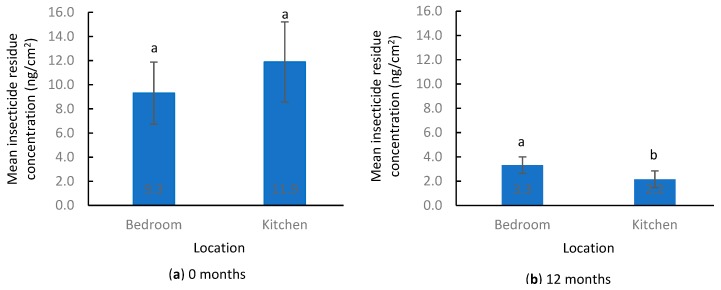
Insecticide residue concentration in settled floor dust before and after IPM program implementation. (**a**) 0 months, (**b**) 12 months. Different lowercase letters above the bars indicate significant differences between room type (Wilcoxon signed-rank test, *p* < 0.05).

Among those apartments that were sampled both at 0 and 12 months, the mean number of insecticides in bedrooms (1.7 ± 0.2) was similar to that in kitchens (1.2 ± 0.2) (Wilcoxon signed-rank test, S = 125.5, *p* = 0.06) (Figure 4a). The mean number of insecticides detected per apartment was 2.5 ± 0.2. When all detected insecticides in each apartment were combined ((bedroom + kitchen)/2), the mean total insecticide residue concentration per apartment was 10.6 ± 2.4 ng/cm^2^. Residue levels in the bedroom were similar to those in the kitchen (Wilcoxon signed-rank test, S = 68.5, *p* = 0.51) (Figure 5a).

### 3.2. Effectiveness of the 12 month Long IPM Program

Among the 49 cockroach-infested apartments that were sampled twice for insecticide residues, the average amount of insecticides applied in each apartment were Advion—32 ± 4 g, Alpine—4 ± 1 g, Maxforce FC Select—2 ± 0 g, Avert—1 ± 0 g, boric acid dust—4 ± 1 g. At 12 months, only one apartment still had cockroaches (total trap count of 4 cockroaches), indicating the IPM program, utilizing baits, was highly successful in eliminating most of the German cockroach infestations. Seven apartments no longer had detectable insecticide residues.

### 3.3. Distribution of Insecticide Residues after IPM Program Implementation

The top two most frequently detected insecticides and their frequency of detection in rooms were cypermethrin (24%) and permethrin (22%) (Figure 2). Compared to 0 months, only cyfluthrin had increased detection frequency. MGK-624 and phenothrin were detected at the same frequency at both periods. All other insecticides had reduced detection frequency or were no longer detected. The mean number of insecticides in the bedroom (1.2 ± 0.2) was significantly greater than that in the kitchen (0.5 ± 0.1) (Figure 4b) (t = 243, *p* < 0.001). Compared to those at 0 months, the mean number of insecticides in bedrooms was similar (Wilcoxon signed-rank test, S = 83, *p* = 0.06), but the number of insecticides in kitchens decreased significantly (Wilcoxon signed-rank test, S = 147, *p* < 0.001). The mean number of insecticides detected per apartment was 1.5 ± 0.2, which was also significantly lower compared to that at 0 months (Wilcoxon signed-rank test, S = 241, *p* < 0.001).

Mean total insecticide residue concentrations also decreased significantly in bedrooms (Wilcoxon signed-rank test, S = 343, *p* < 0.001) and kitchens (Wilcoxon signed-rank test, S = 262, *p* < 0.001). The insecticide residue concentration in bedrooms and kitchens decreased by 64% and 82%, respectively. Bedrooms had significantly higher residue level than kitchens (Wilcoxon signed-rank test, S = 258, *p* = 0.001) (Figure 5b). The mean insecticide residue concentration per apartment was 2.7 ± 0.6 ng/cm^2^, which was 74% lower compared to that at 0 months (Wilcoxon signed-rank test, S = 567, *p* < 0.001).

### 3.4. Effect of Indoxacarb Gel Bait Application on Indoxacarb Residue Concentration

Among the cockroach baits used in the 49 apartments that sampled both at 0 and 12 months, indoxacarb gel bait represented 83% of the total bait usage. Indoxacarb was detectable in 10 apartments at 0 months. Among them, only one apartment had indoxacarb detected in the bedroom. At 12 months, even though an average of 32 g indoxacarb gel bait was applied in each apartment, only two apartments had detectable levels of indoxacarb and one of them did not have detectable residue at 0 months. Therefore, application of indoxacarb gel bait in kitchens and bathrooms in the 49 apartments did not result in increase of indoxacarb concentration in most of the apartments.

## 4. Discussion

The current study confirms that in cockroach-infested apartments, insecticide residues are prevalent (71% had detectable residues) as a result of professional application of insecticides and self-treatment by residents. As would be expected, apartments with higher cockroach numbers tend to have higher insecticide residues. An IPM program, including the use baits rather than insecticide sprays or total release foggers, is not only highly effective in eliminating German cockroach infestations but can also significantly reduce the number of insecticide residues in apartments. The reduction might have been a result of insecticide degradation, loss of residue through house cleaning [36], and termination of new insecticide applications by residents. The four most commonly found insecticides were all pyrethroids. They correspond to the commonly used sprays by residents [7,32]. Commonly used household insecticides contain pyrethroids such as Hotshot Bedbug and Flea spray (a.i.: λ-cyhalothrin, Chemsico, Division of United Industries Corp. St. Louis, MO, USA), Raid Ant and Roach Killer spray (a.i.: Imiprothrin, permethrin, cypermethrin, S.C. Johnson and Son, Inc. Racine, WI, USA), and JT Eaton Kills Bed Bugs spray (a.i.: Deltamethrin, permethrin, JT Easton & Co., Inc. Twinsburg, OH, USA). Raid brand sprays were the most common products used for controlling cockroaches based on a recent survey of residents [32].

In German cockroach-infested homes, cockroaches typically are found in kitchens and bathrooms where food and water are available. The opposite is true for bed bugs, which tend to be concentrated in bedrooms where the human host sleeps. Only one of the enrolled apartments had an existing bed bug infestation at the beginning of the study. We hypothesized that kitchens had higher insecticide residue concentrations than bedrooms as a result of more insecticide usage in kitchens than bedrooms. Yet, we found similar insecticide concentration between kitchens and bedrooms. This might be due to our measurement of residues from drifted dust in the apartment. However, after a 12-month long IPM program, kitchens had lower numbers of insecticides and lower amounts of insecticide residues than bedrooms. One probable reason is the sampling area in the kitchen might be more frequently swept or mopped by residents.

The IPM program was very successful in eliminating most of the German cockroach infestations. Only one of the treated apartments remained infested after 12 months. An average of 18 g of bait was applied per treated apartment. Researchers applied a much higher quantity of bait compared to what had been applied by the contractor prior to the study. The success of the IPM program was also partially due to targeted application of baits guided by trap counts to maximize effectiveness. Follow-up monitoring using traps avoided early termination of treatment and unnecessary treatments in apartments where cockroaches were no longer present. One concern is that while residents reduced their use of pyrethroids and other sprays, the IPM program introduced new insecticides (gel baits) into the apartments. Surprisingly, only two apartments had increased indoxacarb residues from 0 to 12 months. Among the 10 apartments with detectable indoxacarb at 0 months, nine apartments no longer had detectable indoxacarb, despite the fact that all of them received indoxacarb bait. It is known that cockroaches can translocate indoxacarb in the gel bait through excretion or necrophagy [37]. The current study proved that most of the Advion gel bait remained at the application location (cracks, holes, corners of cabinets, etc.) and indoxacarb translocation by cockroaches is negligible. Similarly, application of fipronil-based gel baits (51–99 g of 0.01% fipronil per apartment) in apartments did not result in detectable levels of fipronil in swab samples taken from floors, walls, kitchen counter, and inside cabinets in kitchens at one month after initial bait application [28]. In our study, gel baits containing dinotefuran and fipronil only represented 11% and 4% of the total bait usage compared to 83% indoxacarb gel bait usage in the current study. It would be logical to believe these two insecticides would likely to be undetectable from the floor dust samples because of the physical properties of the gel formulation and the places they were applied. Abamectin is likely to be found in floor wipe samples if measured. However, Avert flowable bait only represented 2% of the total bait usage among the 49 apartments sampled both at 0 and 12months.

Chlorpyrifos, a frequently detected organophosphate insecticide that was reported in homes in the 1990s and early 2000s [38,39], was not found in this study. This is likely due to the ban on indoor use of chlorpyrifos by U.S. Environmental Protection Agency in 2000 (https://www.epa.gov/ingredients-used-pesticide-products/chlorpyrifos#actions). Permethrin is the most frequently detected insecticide in this study. It was also the pyrethroid insecticide most frequently detected in window wipe samples in California homes [40], carpet dust in homes from four states in the U.S. during 1999–2001 [15], and dust samples in daycare centers in 2001 [18]. Cypermethrin was the second most frequently detected insecticide in this study. Concentrations of both permethrin and cypermethrin were still well above detection levels at 12 months, suggesting additional measures must be taken to further reduce insecticide residues in homes.

Residents primarily use insecticide sprays to control cockroaches despite the availability of more effective baits and non-chemical methods are also important to eliminate cockroaches [6]. Parallel to this study, we surveyed another apartment building managed by the same housing authority which had similar levels of pest infestations and was serviced by the same pest control contractor. Face-to-face interviews revealed that 51% of the 81 interviewed residents used insecticide sprays in the past 6 months for controlling cockroaches (Wang et al., unpublished data). Insecticide sprays tend to be applied to exposed areas such as baseboards, the surface of cabinets, walls, etc. Total release foggers were also frequently used. Not only do these methods leave a large number of residues to which occupants can be exposed, but also they are ineffective for eliminating cockroach infestations [28,41]. The current study along with Zha et al. (2018) provides strong evidence that adopting IPM programs and utilizing gel baits for cockroach management will achieve much better cockroach elimination results, as well as significant reduction in indoor insecticide residues [32].

There were limitations to this study. First, we did not record the residents’ insecticide use information prior to taking the dust samples. Obtaining accurate data on changes in insecticide use patterns would allow for interpreting the causes underlying the changes in insecticide residues. The 2nd limitation is a non-intervention control was not included. This was partially out of concern that if we left half of the building untreated while visiting the building very often (every two weeks or monthly), it would cause resident complaints. It was also based on the assumption that cockroach infestation levels and residents’ insecticide use patterns would remain similar from 0 to 12 months if the community continued to use the same pest control services. Nevertheless, this study design would not undermine the conclusions about the impact of a building-wide IPM program on reduction of insecticide residues.

## 5. Conclusions

In cockroach-infested apartments, the majority of the apartments had detectable insecticide residues, with concentrations closely correlated with the cockroach infestation. Permethrin is the most frequently detected insecticide. Bedrooms had similar level of insecticide residues as kitchens based on floor wipe samples. The most commonly found insecticides in apartments reflected the common consumer pesticides used for residential pest control, suggesting they were applied by residents. Changing the cockroach control practice from application of pyrethroid sprays or total release foggers by residents and professionals to IPM with gel bait as the primary control material significantly reduced insecticide residue concentration and number of detectable insecticides. Translocation of indoxacarb in apartments after application of indoxacarb gel bait for cockroach control was minimal and did not result in detectable indoxacarb residues from floor dust samples in most of the sampled apartments.

## Supplementary Materials

The following are available online at https://www.mdpi.com/2075-4450/10/9/304/s1, Quality Assurance/ Quality Control. Table S1: Purity of insecticide analytical standards used in the study, Table S2: Recovery of insecticide analytes and two surrogates from spiked samples, Table S3: Changes of insecticide residue level in apartments from 2016 to 2017.
