# Correction: Wang, C.; et al. Changes in Indoor Insecticide Residue Levels after Adopting an Integrated Pest Management Program to Control German Cockroach Infestations in an Apartment Building. *Insects* 2019, *10*, 304

**DOI:** 10.3390/insects10110406

**Published:** 2019-11-15

**Authors:** Changlu Wang, Amanda Eiden, Richard Cooper, Chen Zha, Desen Wang, Ed Reilly

**Affiliations:** 1 Department of Entomology, Rutgers University, New Brunswick, NJ 08901, USA; amandalynneiden@gmail.com (A.E.); rick.cooper@cooperpest.com (R.C.); zcfustc@gmail.com (C.Z.); 2 Key Laboratory of Bio-Pesticide Innovation and Application of Guangdong Province, Department of Entomology, College of Agriculture, South China Agricultural University, Guangzhou 510642, China; desen@scau.edu.cn; 3 New Jersey Department of Environmental Protection, Trenton, NJ 08625, USA; Ed.Reilly@dep.nj.gov

Following the publication of our article [1], we found that an incorrect data set was used in the regression analysis. Figure 1 and the associated analysis results need to be changed.

The corrected Figure 1 and Figure 1 legend are listed below:

On page 5 (line 2–4), the sentence is changed to the following: “Insecticide residue concentration in apartments was not correlated with the cockroach counts recorded by the cockroach traps (F = 0.38; df = 1, 25; *p* = 0.54, R^2^ = 0.02) (Figure 1)”.

On page 9, line 2, within Section 5, “Conclusions”, the following phrase is deleted: “with concentrations closely correlated with the cockroach infestation”.

The authors wish to apologize for the mistake made and inconveniences caused.

## Figures and Tables

**Figure 1:**
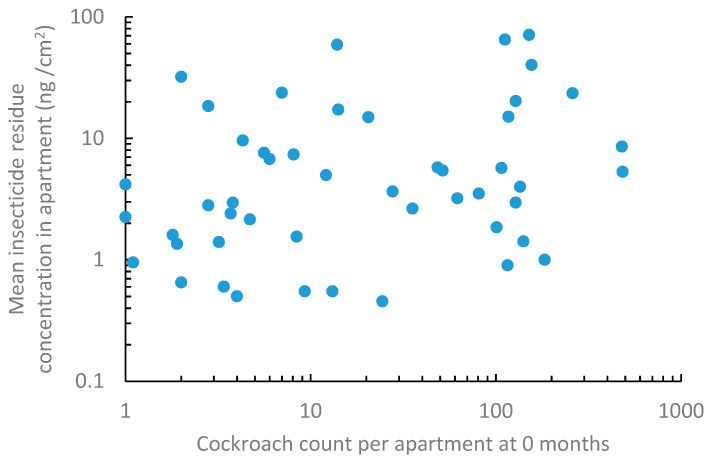
Association between insecticide residue concentration per apartment (mean residue concentration measured in bedroom and kitchen) and initial cockroach count. For regression analysis, those apartments without detected residue and those with a cockroach count <10 were excluded because there are many apartments with small counts, which cause the data to be not normal even after logarithmic transformation.
