# Supplementary material for: Changes in Indoor Insecticide Residue Levels after Adopting an Integrated Pest Management Program to Control German Cockroach Infestations in an Apartment Building
Source: Insects. 2019 Sep 18;10(9):304. doi: 10.3390/insects10090304 (PMC6780151; doi:10.3390/insects10090304)
Supplement: Supplementary file 1 [file insects-10-00304-s001.pdf]

Supplemental Material

Changes in Indoor Insecticide Residue Levels after Adopting an Integrated Pest Management Program to Control German Cockroach Infestations in an Apartment Building

Changlu Wang,*[1] Amanda Eiden,[1] Richard Cooper,[1] Chen Zha,[1] Desen Wang,[2] and Ed Reilly[3]

* Corresponding author. E-mail: changluw@rutgers.edu.

[1] Department of Entomology, Rutgers University, New Brunswick, NJ 08901.

[2] Key Laboratory of Bio-Pesticide Innovation and Application of Guangdong Province, Department of Entomology, College of Agriculture, South China Agricultural University, Guangzhou 510642, Guangdong Province, China

[3] New Jersey Department of Environmental Protection, Trenton, NJ 08625

Quality Assurance/ Quality Control

All samples in this study were spiked with two surrogates to monitor both the extraction and analysis efficiency. Dibutyl chlorendate and triphenyl phosphate were the two surrogates added to each sample at approximately 12 μg each. Laboratory blanks and matrix blanks were analyzed along with the surface wipe samples as controls. Sample spikes consisting of all the insecticide analytes at eight different concentration levels throughout the working range were used to determine the recovery of each insecticide. The insecticide neat standards were obtained from the EPA pesticide repository located in Fort Meade Maryland. Purity of the analytical standards are shown in Table S1. Stock standards were prepared from the neat standards at a concentration of approximately 400 ug/ml in ethyl acetate. Intermediate insecticide standard mixtures were prepared from the individual stock standards at a concentration of approximately 40 ug/ml for each insecticide analyte. The amounts added for each insecticide in the eight concentration levels were as follows: 0.2 μg, 0.4 μg, 0.6 μg, 0.8 μg, 1.0 μg, 2.0 μg, 4.0 μg, 5.0 μg. Since pyrethrin is a multi-component insecticide analyte, higher spike concentrations were used. The eight concentration levels for pyrethrin were as follows: 1.0 μg, 2.0 μg, 3.0 μg, 4.0 μg, 5.0 μg, 10 μg, 20 μg, 25 μg. The average recovery and relative standard deviation for each insecticide analyte based on thirty-two spiked samples can be found in Table S2. The average recovery and relative standard deviation for the two surrogates which were based on 309 samples can also be found in Table S2.

The sample analysis utilized an external standard technique with calibration curves consisting of six concentration points for insecticide analyte and a linear regression algorithm was used for quantification. The method and instrument detection limits are also listed for each insecticide analyte in the table above. EPA pesticide external standard methods for water samples such as method 608 was used as a model for the analysis of the swab samples.

The extraction procedure is like surface wipe extractions performed by Federal Insecticide Fungicide Rodenticide Act (FIFRA) state laboratories for pesticide enforcement cases. The United States Environmental Protection Agency Office of Enforcement and Compliance Monitoring generated the "NEIC Pesticide Sampling Guide" which was used as a model for this study. This guide was generated in August 1985 by Robert F. Schneider for the National Enforcement Investigations Center which was in Denver, Colorado.

Qualitative identification of insecticide analytes in samples were confirmed when the relative abundance of characteristic ions in the mass spectrum of both the standard and the sample agreed

within 20% absolute abundance. For example, if a characteristic ion has a relative abundance of 30% in the standard spectrum, its abundance in the sample spectrum must be in the range of 10 – 50%. In this study there were no qualitative identification of insecticide analytes below the method detection limits (MDL) and if insecticide analytes were qualitatively confirmed below the MDL the quantified value would be reported as the MDL since quantifying in this region would be beyond the linear range.

The MDL was determined by spiking eight blank swab samples with approximately 40 ng of each insecticide analyte for a final concentration of approximately 0.22 ng/cm^2. The eight spiked samples were analyzed and the standard deviation was determined for each insecticide analyte. Since there were 7 degrees of freedom from the student t test the standard deviation for each insecticide analyte were multiplied by 2.998 to generate the MDL values. The instrument detection limits were determined for each insecticide analyte by obtaining the peak height of three times the signal to noise level and comparing it to the peak height of the lowest concentration data point in the calibration curve to generate the value.

The column pressure setpoint was adjusted to 6894.7 pascals at a constant flow rate setting to produce a linear velocity of 100 cm/sec and a flow rate of 13.2 ml/min. The injector temperature was set to 523ºK and a splitless injection was used with a split vent of 50ml/min at 0.7min. The mass spectrometer ion source was set at 503ºK and the temperature of the quadrupole was 423ºK. The transfer line leading to the mass spectrometer was set at 883ºK. Elution of the insecticide analytes from the GC column occurred through the temperature program that ranged from 323ºK to 573ºK over a 7-minute chromatographic run.

Table 1. Purity of insecticide analytical standards used in the study.

Insecticide Standard	2016		2017	
	**Lot Number**	**Purity**	**Lot Number**	**Purity**
Chlorfenapyr	L76-56	0.996	L76-56	0.996
Chlorpyrifos	TSN100227	0.998	IWS-80227-47	1
Fipronil	52200301	0.991	522200301	0.991
Indoxacarb	JW 062 181	0.996	JW 062 181	0.996
Pyriproxyfen	A51723b	0.998	AS1723b	0.997
Pyrethrin	AA8729	0.194	AA8729	0.194
Allethrin	C150523	1	C150523	1
Cyfluthrin	208200502	0.492	208200502	0.492
Cypermethrin	SZBC047XV	0.943	575597	0.994
Deltamethrin	23983	0.936	902200301	0.994
Esfenvalerate	B656-058 0035 739	0.987	WS-S-FV-07001	0.986
Imiprothrin	C151029	0.991	C151029	0.991
Permethrin	713200501	0.484	410559	0.997
Phenothrin	C150325	1	C150325	1
Resmethrin	1029200311	0.998	1029200311	0.998
Tetramethrin	23983	0.936	23983	0.936
λ-Cyhalothrin	4230400	0.995	4230400	0.995
MGK-264	AB4552	0.952	AB4552	0.952

Table 2. Recovery of insecticide analytes and two surrogates from spiked samples.

Spiked Analytes	Average Recovery	Relative Standard Deviation	Method Detection Limit (ng/cm²)	Instrument Detection Limit (ng/cm²)
Chlorfenapyr	88.90%	15.40%	0.66	0.039
Chlorpyrifos	81.90%	14.10%	0.71	0.05
Fipronil	83.30%	22.40%	0.93	0.14
Indoxacarb	91.80%	10.90%	0.49	0.13
Pyriproxyfen	87.40%	20.70%	0.9	0.016
Pyrethrin	89.90%	14.50%	0.83	0.18
Allethrin	83.60%	12.20%	0.55	0.031
Cyfluthrin	92.20%	17.20%	0.5	0.16
Cypermethrin	94.50%	15.80%	0.5	0.15
Deltamethrin	94.50%	13.90%	0.43	0.13
Esfenvalerate	87.70%	15.70%	0.71	0.13
Imiprothrin	103%	21.00%	0.89	0.065
Permethrin	89.70%	17.10%	0.61	0.044
Phenothrin	85.70%	18.00%	0.68	0.028
Resmethrin	81.30%	9.31%	0.46	0.031
Tetramethrin	85.00%	14.50%	0.49	0.042
ʎ-Cyhalothrin	83.10%	22.70%	1.1	0.051
MGK-264	84.60%	11.80%	0.43	0.047
Dibutyl Chlorendate	84.40%	11.40%		
Triphenyl phosphate	89.70%	15.60%		

Table 3. Changes of insecticide residue level in apartments from 2016 to 2017.

Insecticide	n	Mean Change (ng/cm^2)	Standard Error (ng/cm^2)	Statistics*
Chlorfenapyr	7	7.41	1.47	t = 5.0; P = 0.002
Indoxacarb	12	0.72	2.17	S = 19; P = 0.15
Cyfluthrin	7	-0.57	0.59	t = -0.97, P = 0.37
Cypermethrin	32	5.65	1.89	S = 197, P < 0.0001
Deltamethrin	17	5.19	1.74	S = 64.5, P = 0.001
Imiprothrin	14	10.55	3.09	S = 52.5, P = 0.0001
Permethrin	32	1.4	0.46	S = 198, P < 0.0001
Phenothrin	4	2.03	2.12	t = 0.96, P = 0.41
Tetramethrin	3	27.33	10.93	t = 2.5; P = 0.13
λ-cyhalothrin	20	0.63	0.58	S = 38.5; P = 0.16
MGK-264	13	9.63	4.64	S = 31.5, P = 0.03

* Paired student t test was used for data that are normally distributed. Wilcoxon signed rank test was used for data that are not normally distributed. Non-normally distributed data were not transformed before analysis.
